# Vital Pulp Therapy in Cats: 47 Cases (2009-2025)

**DOI:** 10.1177/08987564261450452

**Published:** 2026-05-20

**Authors:** Lena Norman, Karolina Brunius Enlund, Ewa Chronowska, Daria Ziemann, Jerzy Gawor

**Affiliations:** 1 Department of Clinical Sciences, 8095 Swedish University of Agricultural Sciences, Uppsala, Sweden; 2 Evidensia Djursjukhuset Västerort, Spånga, Sweden; 3 594789 Klinika Weterynaryjna Arka, Kraków, Poland

**Keywords:** vital pulpotomy, VPT, feline dentistry, mineral trioxide aggregate, MTA, complicated crown fracture, veterinary dentistry

## Abstract

Vital pulp therapy (VPT) is a dental procedure aimed at preserving tooth vitality after pulp exposure by removing part of the exposed coronal pulp and placing a protective pulp dressing material directly over the remaining pulp, followed by a tightly sealed restoration to prevent bacterial leakage at the restoration–dentin interface. Indications include recent complicated crown fracture (CCF) or traumatic malocclusion in mature, as well as immature, permanent teeth. This retrospective study evaluated the outcome of 47 teeth in 36 cats treated with VPT at a referral clinic between 2009 and 2025, using either dental radiography, cone beam computed tomography (CBCT) or both modalities. The overall success rate was 81%, supporting VPT as a viable alternative to extraction or root canal therapy in feline teeth with recent CCF or traumatic malocclusion. Although not statistically significant, teeth with CCF and associated contaminated pulps exhibited higher failure rates than teeth in traumatic malocclusion, which were cases that had crown height reduction performed in a more sterile environment, and which had uncontaminated pulp at the time of VPT. Mineral trioxide aggregate was used as the pulp capping material in all cases. Limitations include the retrospective design, limited sample size, and large variation in time from VPT to follow-up. Results emphasize the clinical benefit of VPT in cats, while highlighting the importance of case selection, sufficient follow-up, and further prospective research to optimize protocols and long-term outcomes.

## Introduction

Traumatic injuries to teeth in cats, including complicated crown fracture (CCF) (a fracture of the crown of the tooth that involves the pulp), are common problems in veterinary practice.^1–6^ Malocclusion, traumatic contact between a tooth and the lip or labial mucosa and upper lip entrapment after extraction of maxillary canine teeth, are other pathologies seen in feline veterinary dentistry.^7–9^

A standard approach for many dental conditions is dental extraction.^1^ However, canine teeth are considered to be strategic teeth, contributing to the structural strength of the jaws and a fully functional occlusion, as well as the ability to hold and kill prey.^10^ Dental extraction of canine teeth may cause unnecessary trauma, risk of dehiscence, iatrogenic jaw fracture, oronasal communication, and prolonged time of anesthesia.^1^ In addition, extraction of the maxillary canine teeth in cats can cause upper lip entrapment from the mandibular canine teeth.^11,12^ Root canal therapy has been described and is an accepted treatment modality for select cases in cats, but it is a time-consuming procedure that requires substantial amounts of material and a wide range of instruments. Moreover, the treatment results in a non-vital tooth, which no longer maintains its natural physiological functions.^13,14^

A potential alternative to dental extraction or root canal therapy is vital pulp therapy (VPT), which may be considered a good option in some cases.^13–17^

In human dentistry, VPT is a broader term that encompasses various procedures aimed at preserving pulp vitality in teeth affected by decay, trauma, or other factors. It includes techniques such as indirect pulp capping, direct pulp capping, miniature pulpotomy, partial pulpotomy, full pulpotomy, and partial pulpectomy.^18,19^ In veterinary dentistry, VPT primarily refers to the procedure involving a partial pulpotomy and application of a bioactive material (eg, mineral trioxide aggregate [MTA]), glass ionomer, and composite to preserve vitality of the tooth.^15^

Potential complications of VPT include: pulpitis, pulp necrosis and periapical pathology, therefore regular follow-up is recommended.^16,20^ Success may be evaluated by dental radiography and/or cone beam computed tomography (CBCT), where vitality of the tooth as well as potentially observed radiographic abnormalities are examined.^21^

As veterinary medicine and dentistry continue to evolve, with growing demand for effective dental care methods, it is important to keep providing the best care possible for both feline and canine patients. To the authors’ knowledge there are few scientific studies regarding the outcome of VPT in cats.^16,22^ Although some studies have addressed VPT in dogs,^15–17^^,23^ cats require more targeted studies to ensure effective treatment and to advance feline dental care. The aim of this study was to investigate the success rate of VPT in cats and to evaluate factors associated with its outcome.

## Materials and Methods

### Case Selection

Medical records of cats that underwent VPT between May 2009 and March 2025 at a European Veterinary Dental College (EVDC) approved training center were reviewed. Inclusion criteria consisted of complete anesthesia and dental records, and at least 1 follow-up examination with available intraoral radiographs and/or CBCT images that contained radiographs/CBCT images before, immediately after and during the last available examination following VPT. Where multiple follow-ups were available, only the latest was used for this study.

### Data Collection

Variables recorded included signalment of the cat (age, breed, sex, body weight), affected tooth, time from VPT to latest follow-up, diagnostic imaging modality (dental radiography, CBCT or both), indication for VPT, status of the pulp at VPT (intact or contaminated). Contaminated pulp was defined as exposure of the pulp at any time before arrival at the clinic. Interval between pulp exposure and treatment in CCF cases, pulp dressing material, intraoperative pulpal hemorrhage, and administration of antimicrobial agents were also documented.

### Vital Pulp Therapy Procedure

VPT was performed according to accepted principles of human dentistry,^20,24^ with modifications for feline dental anatomy.^13^ All treatments were performed by residents of the EVDC under supervision of a EVDC Diplomate (JG).

For CCF cases, VPT was preferred when treatment occurred within 48 hours, but accepted up to 96 hours after pulp exposure. For traumatic malocclusion cases, VPT was performed immediately upon pulp exposure by crown height reduction. Cats with systemic disease (eg, renal or cardiac insufficiency) were not eligible for the procedure.

**Figure 1.**
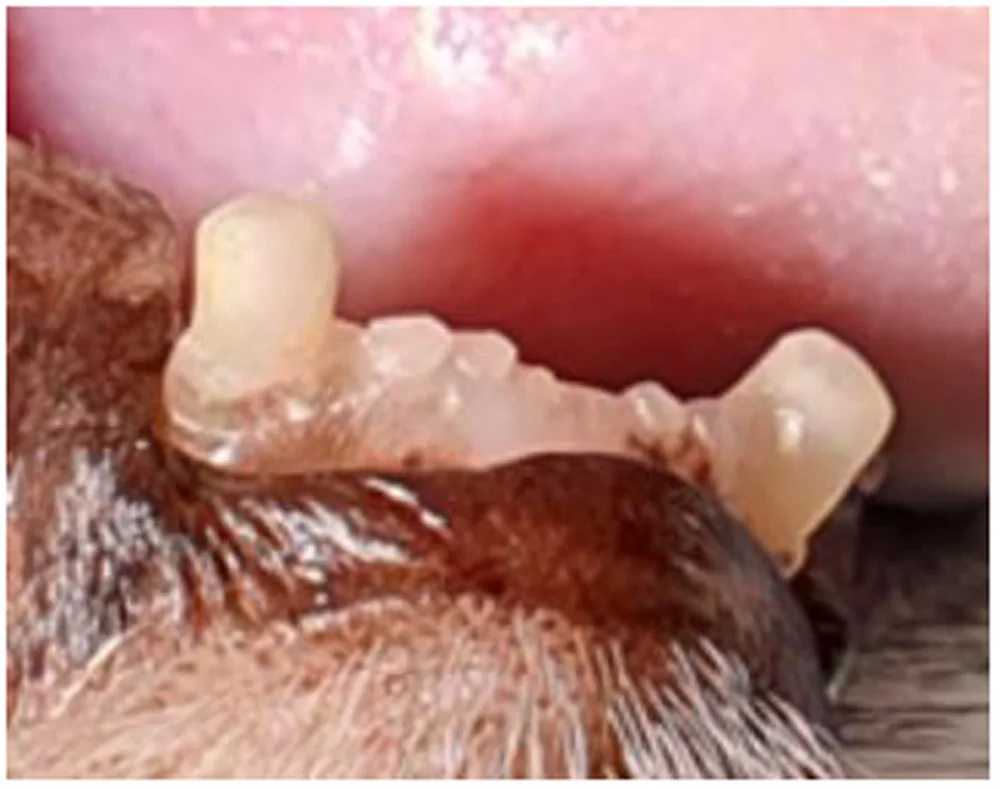
Photograph of the right and left mandibular canine teeth immediately after crown height reduction and vital pulp therapy in a cat.

All procedures were performed under general anesthesia. Prior to surgery all teeth were ultrasonically scaled and polished using fluoride-free pumice, isolated with a rubber dam, and prepared under aseptic conditions. Coronal pulp tissue (2-4 mm) was removed using a sterile, round #9 diamond bur^a^ on a high-speed handpiece that was cooled with distilled water. Hemostasis was achieved using 0.9% saline-moistened paper points^b^. The exposed pulps were dressed with 1 to 2 mm of MTA^c^, followed by 0.5 to 1 mm of glass ionomer^d^, which served as an intermediate restoration layer. After etching and bonding, a 0.5 to 1 mm layer of hybrid composite restorative material^e^ was used to fill the remaining part of the coronal pulp chamber. The surface of the restoration was smoothed using sanding discs^f^. Finally, a layer of unfilled resin^g^ was applied to reduce the risk of marginal leakage (Figure 1).

Perioperative analgesia included a regional nerve block using lidocaine^h^ (20 mg/mL) and postoperative meloxicam^i^ (0.05 mg/kg) given once daily PO, and buprenorphine^j^ (10-30 µg/kg) as a single intramuscular injection. The minimum length of pain management protocol with meloxicam was 5 days after treatment. The decision to use, or not to use, antibiotics was determined by clinician preference based on overall patient condition and CBC results.

### Radiographic Evaluation

Preoperative, immediate postoperative, and follow-up intraoral radiographs and/or CBCT images were used in this study. Dental radiographs were obtained using either a phosphoric plate^k^ in digital systems^l^ or a direct digital sensor with imaging software^m^. High-resolution 3D diagnostic imaging was obtained using full-mouth CBCT^n^. The CBCT scans were analyzed with the use of NNT viewer software^o^ provided by the manufacturer.

Restoration quality was evaluated clinically and radiographically immediately after and at the last available follow-up examination.

Follow-up recommendations were for examinations at 3 and 12 months post-VPT, and annually thereafter.

Radiographs and CBCT images were evaluated by JG. In cases where both CBCT images and dental radiographs were available from the last follow-up, they were both evaluated, and a consensus of the results was used in the study. Radiographic evaluation included evidence of tertiary dentin bridge and secondary dentin formation, apical closure (when applicable), periapical status and the presence or development of tooth resorption (TR).

Outcome criteria followed the guidelines of the European Society of Endodontology^20^ except for response to pulp sensitivity tests and absence of pain due to the difficulty of determining that in the feline patient^25,26^ as well as tertiary dentin bridge formation, which was recorded, but not required for a classification of success.^27^

Success was defined as occurrence of secondary dentin formation, apical closure (when applicable) and the absence of clinical and radiographic signs of internal root resorption and apical periodontitis.

Cases with no evidence of secondary dentin formation at early follow-up after VPT, and those where mild ankylosis was seen at the time of VPT, but the ankylosis did not progress, were also considered successful if all other criteria for success were fulfilled.

Treatment was considered to have failed when secondary dentin deposition ceased, root formation in immature teeth halted, the restoration was missing, periapical lesions developed, or TR developed.

### Statistical Analysis

The probability of treatment success was analyzed using generalized linear mixed models with a binomial distribution and logit link, including patient ID as a random intercept to account for multiple observations on the same individual. Three models were investigated. Firstly, including all data, the probability of success was modeled using time to latest examination, the individual's age and their pulp status as independent variables. This was followed by a model only on individuals with a contaminated pulp. In this model, the probability of success was modeled using time to treatment as an independent variable. An additional model was analyzed for all observations, modeling the probability of success by whether the apex was open or closed prior to surgery as an independent variable.

## Results

Between 2009 and 2025, a total of 112 teeth in 92 cats underwent VPT at an EVDC approved training center under supervision of an EVDC Diplomate. Of these, 47 teeth (42%) in 36 cats (39%) had at least 1 follow-up examination using intraoral radiography and/or CBCT and were included in the study. Eleven cats underwent VPT on 2 teeth during the same procedure.

### Study Population

The study population consisted of 24 Domestic Shorthaired, 2 Domestic Longhaired, 2 Norwegian Forest, 3 British Shorthaired, 2 Oriental, 1 Burmese, 1 British Longhaired, and 1 Devon Rex cat. The sex and reproductive status distribution consisted of intact females (*n* = 5), spayed females (*n* = 15), intact males (*n* = 5), and castrated males (*n* = 11). Body weights ranged between 3.3 and 7.0 kg, mean weight was 4.5 kg. Ages ranged from 4 months to 19 years. Distribution of age groups is presented in Table 1.

**Table 1.** Age Distribution of Cats (N = 36) Included in Follow-Up After Vital Pulp Therapy.

Age Group (Years)	Cats (*n*)	%
≤1	10	28%
2-3	5	14%
4-5	6	17%
6-7	7	19%
8-9	3	8%
10-11	2	6%
12-13	2	6%
19	1	3%

### Treated Teeth

VPT was performed primarily on canine teeth (Table 2). Exceptions included 1 maxillary fourth premolar and 1 mandibular fourth premolar.

**Table 2.** Distribution of Teeth Treated With Vital Pulp Therapy (N = 47) in Cats (N = 36).

Tooth	n	%
104 (Right Maxillary Canine)	7	15%
108 (Right Maxillary Fourth Premolar)	1	2%
204 (Left Maxillary Canine)	8	17%
304 (Left Mandibular Canine)	16	34%
404 (Right Mandibular Canine)	14	30%
408 (Right Mandibular Fourth Premolar)	1	2%

### Indications, Intraoperative Findings, and Treatment

Indications for VPT included CCF (*n* = 21), traumatic contact between a tooth and the lip or labial mucosa (*n* = 4)—of which 2 were seen after maxillectomy and the other 2 were complications/upper lip entrapment after extraction of a maxillary canine tooth, malocclusion (*n* = 8), and prophylaxis to prevent traumatic contact between a tooth and the lip or labial mucosa (*n* = 14)—of which 2 were done at the time of maxillectomy and 2 were done at the time of extracting an impacted maxillary canine tooth whereas the remaining 10 were done at the time of extraction of a maxillary canine tooth.

At the time of treatment, 26 teeth had intact crowns with no pulp exposure, and 21 had fractured crowns with exposed and contaminated pulp. Of the teeth with CCF, 9 received treatment within 48 hours, while 12 were treated 60 to 96 hours postfracture.

No excessive intraoperative pulpal hemorrhage was recorded in any of the teeth treated with VPT.

### Follow-Up and Imaging

Time from VPT to last follow-up ranged from 28 to 3444 days (mean 606 days) postoperatively. Out of the 36 cats in the study, 8 cats (22%) had a follow-up interval within 3 months, 10 (28%) between 3 and <12 months, 10 (28%) between 12 and 36 months, and 8 (22%) at >36 months.

Out of the 47 teeth in 36 cats in the study, 37 teeth in 28 cats had only 1 follow-up, 8 teeth in 6 cats had 2 follow-ups, and 2 teeth in 2 cats had 3 follow-ups. Imaging modality included dental radiography alone (*n* = 31), CBCT alone (*n* = 8), or a combination of both modalities (*n* = 8).

### Antibiotic Use

All but 2 cats, representing 3 treated teeth, were administered systemic antibiotics. Cefovecin^p^ alone was used most frequently (*n* = 24). One of the 2 cats that did not receive antibiotics had VPT performed on 2 teeth of which 1 was deemed successful and the other 1 a failure.

### Restoration Quality

All restorations were clinically intact and smooth at the time of surgery. At follow-up, restorations were assessed as intact in 42 teeth, absent in 3, and rough in 2 teeth. Radiographic voids within the restorative material were detected immediately after treatment in 11 teeth. None of the 5 teeth with later missing or rough restorations had shown voids initially. Restoration length was categorized as short (2 mm; *n* = 21) or standard (3-4 mm; *n* = 26).

### Radiographic Outcomes

**Figure 2.**
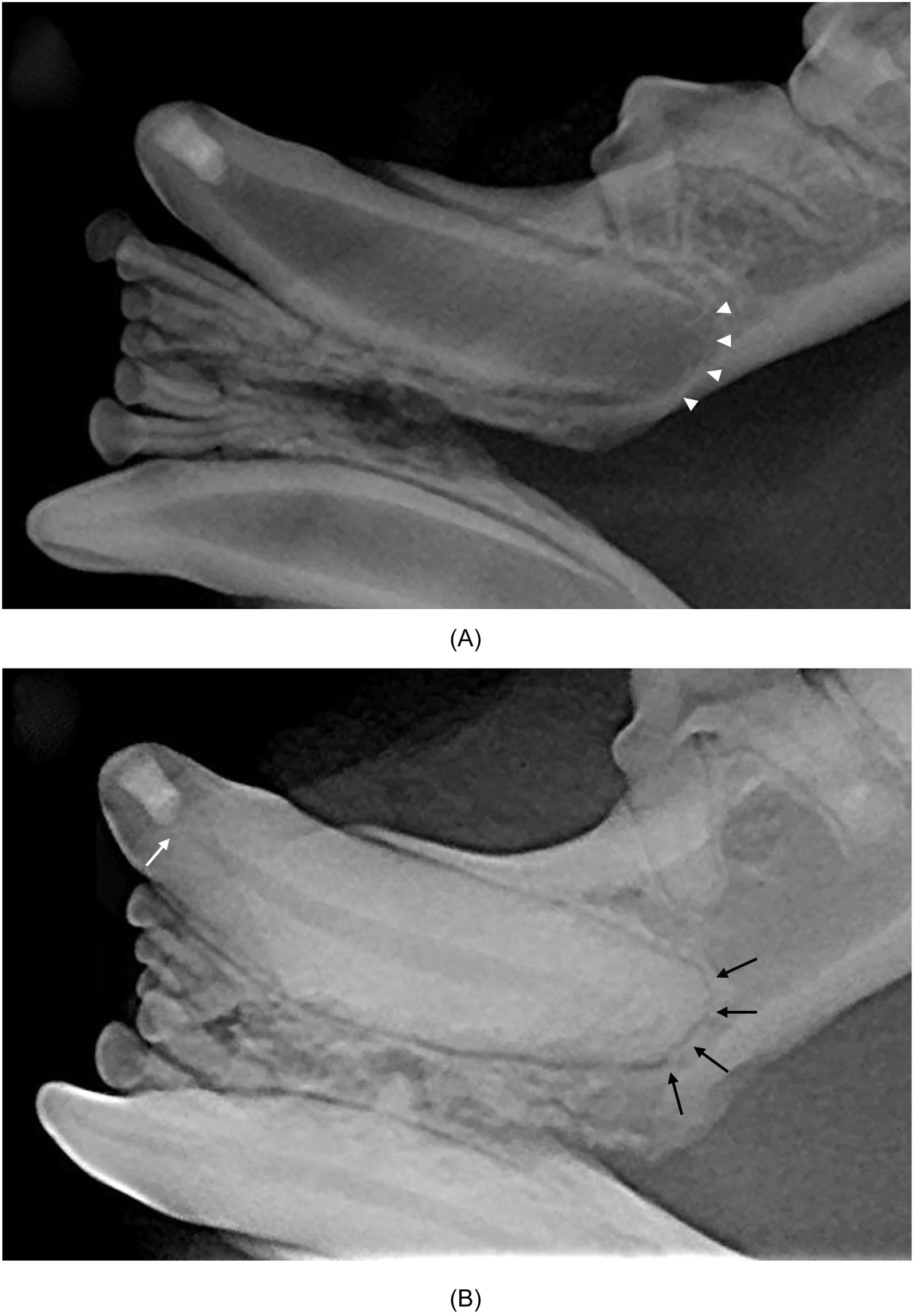
Radiographic views obtained (A) immediately after vital pulp therapy and (B) at the 2-year follow-up showing successful treatment in a 6-month-old cat. Note the open apex (white arrowheads) in image (A) and the closure of the apex (black arrows), tertiary dentin bridge formation (white arrow), progressive narrowing of the pulp cavity, and absence of periapical radiolucency in image (B).

**Figure 3.**
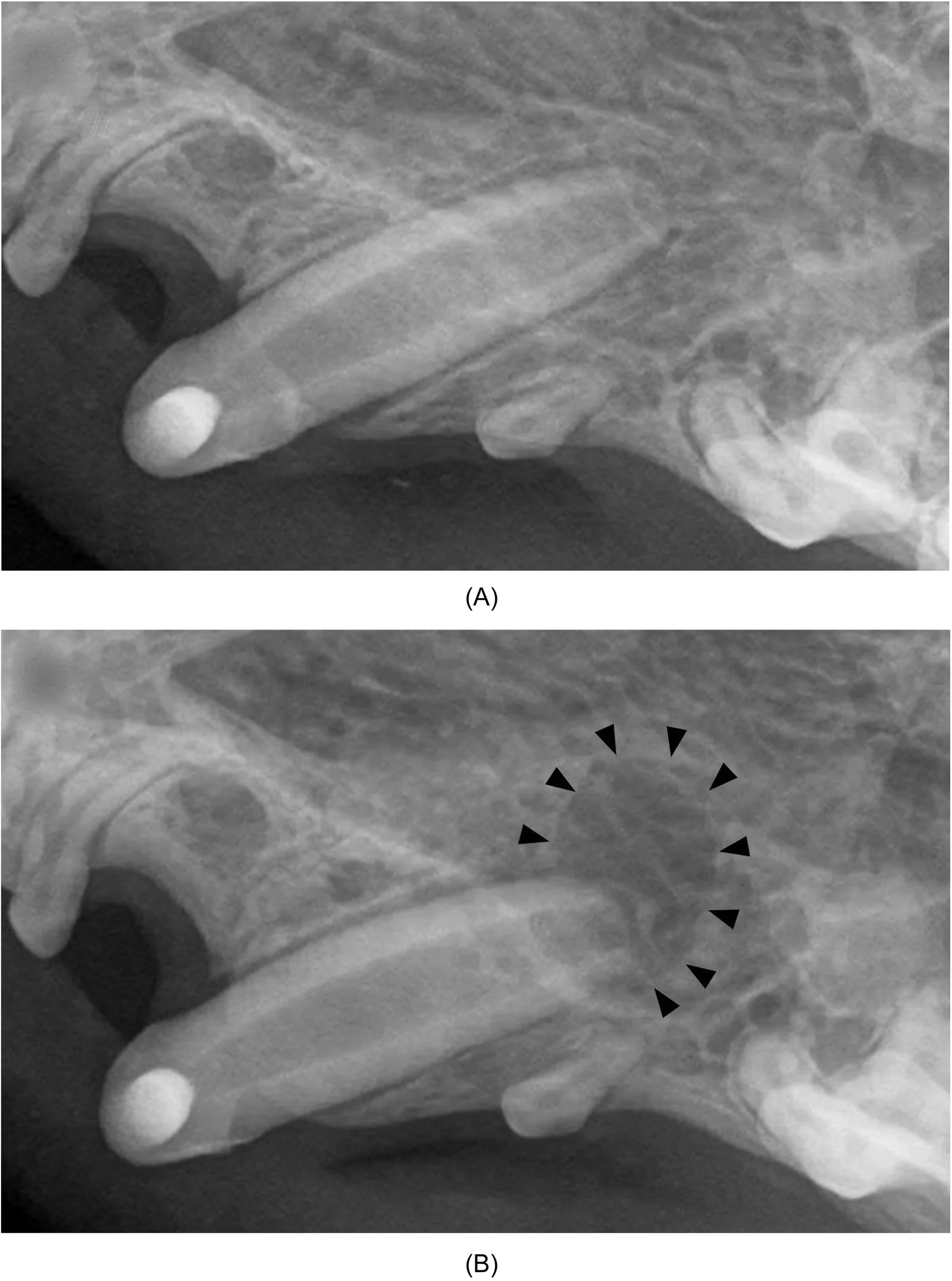
Radiographic views obtained (A) immediately after vital pulp therapy and (B) at the 3-month follow-up showing failed treatment in an 8-month-old cat. Note the well-defined periapical radiolucency (black arrowheads) in image (B).

Radiographic evidence of dentin bridge formation was identified in 39 teeth (83%) (4/9 in the failure group and 35/38 in the success group). Secondary dentin deposition was evident in 36 teeth (77%). In 6 cases where secondary dentin was not yet visible, all were nonetheless judged to represent successful outcomes because follow-up times were short (28-67 days) and no other clinical or radiographic pathology was present. Among the 10 teeth with open apices at the time of surgery, 7 demonstrated radiographic apical closure at the time of follow-up (Figure 2). At follow-up, 5 teeth, including the 3 teeth with open apices at the time of VPT, showed evidence of apical periodontitis, defined as an apical periodontal ligament width more than twice that observed elsewhere in the same tooth (Figure 3). There were no signs of TR in 39 teeth both initially and at follow-up. Six teeth showed mild ankylosis prior to VPT, which was unchanged during follow-up. One tooth developed TR type 1 and was extracted. Another tooth developed TR type 2 and underwent crown amputation.

### Success and Failure

Of the 47 treated teeth, 38 teeth (81%) in 28 cats were classified as successful and 9 teeth (19%) in 8 cats as failed (Table 3). Causes of failure included apical periodontitis (*n* = 5), restoration loss (*n* = 2), and TR (*n* = 2; type 1 and type 2). Of the failed teeth, 4 were extracted, 1 underwent crown amputation, 2 received root canal therapy, and 2 underwent apexification followed by root canal therapy. Failure was most frequently associated with CCF and contaminated pulps (7/9, 78%). Of these, 4 were treated within 48 hours, and 3 were treated within 72 to 96 hours of trauma. Follow-up intervals in failed cases ranged from 1 month to 4 years (35-1509 days). Early failures (5 teeth in 4 cats) occurred between 1 and 4 months (35-118 days) after VPT. Late failures (4 teeth in 4 cats) were seen between 8 months and 4 years (252-1509 days) after VPT. Out of the 4 cats with late failures, 1 cat had 3 follow-ups over the course of 3 years, and 1 cat had 2 follow-ups over the course of 4 years. The third cat with a late failure was 19 years old when VPT was completed and follow-up was performed 2.5 years later. The fourth cat with late failure had a follow-up 8 months after VPT.

**Table 3.** Overview of Outcomes Stratified by Clinical and Radiographic Features of Teeth Treated With Vital Pulp Therapy (N = 47) in Cats.

Parameter	Success (*n* = 38; 81%)	Failure (*n* = 9; 19%)
Pulp status at treatment	37% (*n* = 14) contaminated 63% (*n* = 24) intact	78% (*n* = 7) contaminated 22% (*n* = 2) intact
Timing of treatment of contaminated pulps	36% (5/14) teeth ≤48 h; 64% (9/14) teeth at 60-96 h	57% (4/7) teeth ≤48 h; 43% (3/7) teeth at 60-96 h
Restoration status at follow-up	95% (*n* = 36) intact 5% (*n* = 2) rough 0% (*n* = 0) missing	67% (*n* = 6) intact 0% (*n* = 0) rough 33% (*n* = 3) missing
Restoration length	45% (*n* = 17) short (2 mm); 55% (*n* = 21) standard (3-4 mm)	44% (*n* = 4) short (2 mm); 56% (*n* = 5) standard (3-4 mm)
Voids	74% (28/38)	11% (1/9)
Dentin bridge formation	92% (*n* = 35) present; 8% (*n* = 3) absent	44% (*n* = 4) present; 56% (*n* = 5) absent
Secondary dentin formation	84% (*n* = 32) present; 16% (*n* = 6) no evidenced^a^	44% (*n* = 4) present; 56% (*n* = 5) no evidenced
Apical closure	6 open → all closed	4 open → 1 closed
Radiographic pathology	None significant; ankylosis stable	56% (*n* = 5) apical periodontitis; 22% (*n* = 2) TR; 22% (*n* = 2) missing restorations
Tooth distribution	65% (*n* = 25) mandibular canines; 29% (*n* = 11) maxillary canines; 2% (*n* = 1) 108; 2% (*n* = 1) 408	56% (*n* = 5) mandibular canines; 44% (*n* = 4) maxillary canines
Follow-up times	42% (*n* = 16) short-term (1-6 months); 58% (*n* = 22) long-term (≥8 months)	56% (*n* = 5) short-term (1-6 months); 44% (*n* = 4) long-term (≥8 months)

a No evidenced secondary dentin in successes mostly at ≤67-day follow-ups.

Notably, all 3 teeth with missing restorations at follow-up were classified as failures, even though only 1 of these developed apical periodontitis. Only 1 failure corresponded to a tooth that had demonstrated voids in the restoration after treatment, while the 2 teeth with rough restorations at follow-up were categorized as successful outcomes.

Out of the 38 teeth that were deemed successful, 16 teeth were evaluated after 1 to 6 months (28-182 days) and 22 teeth had their latest follow-up 1.0 to 9.4 years (353-3444 days) after VPT. Fourteen of the successfully treated teeth initially had pulp contamination due to CCF, whereas the remainder had intact pulp at VPT. In the successful CCF cases, VPT was performed within 48 hours from trauma in 5 teeth and after 60 to 96 hours from trauma in 9 teeth. Two cats with teeth treated bilaterally demonstrated mixed outcomes (1 tooth was deemed a failure and the other 1 a success).

No statistically significant association between treatment success or failure and age, pulp status (intact or contaminated at the time of VPT), time from CCF to VPT, or time from VPT to latest follow-up was identified in the analysis.

## Discussion

This retrospective study represents, to the authors’ knowledge, the largest evaluation of VPT outcomes in feline patients to date, with 47 teeth in 36 cats followed radiographically and/or with CBCT.

A previous study evaluated the outcome of VPT in 6 canine teeth in cats, all of which were successful, and stressed the necessity of objective radiographic follow-up rather than reliance on owner assessment alone. In the present investigation, an overall success rate of 81% was found, closely mirroring both a study from 2025 which reported 80% VPT success in dogs,^23^ and another from 2018 reporting 81% success following feline root canal therapy,^43^ using similar outcome criteria.

The number of follow-up assessments per cat ranged from 1 to 3, meaning that the extent to which each cat's progress could be monitored over time varied substantially. Late failures, occurring between approximately 8 months and 4 years after VPT, were identified in 4 teeth across 4 cats, illustrating that complications may arise long after initially successful outcomes (Table 3). In the current study, time from VPT to latest follow-up varied widely, ranging from as short as 28 days to as long as 9.4 years. Such a broad follow-up period is beneficial because it allows detection not only of early complications, such as restoration loss or apical periodontitis occurring within the first few months, but also late-onset issues like TR or delayed failure that may manifest several years after treatment. Comparable observations were reported in a study regarding VPT in dogs, where, in the group of dogs that had MTA as dressing material, half of the failures (6/12) occurred within 2 to 11 months following treatment, and the other half were diagnosed more than 12 months postoperatively.^15^ Regular follow-up assessments with dental radiography or CBCT are recommended to ensure early intervention in cases that failed, which could enhance tooth retention and patient quality of life.

Although not statistically significant, the fact that failures were observed across age ranges, breeds, and both sexes suggests that neither patient factors nor the specific tooth were decisive determinants of outcome in this study.

The observation that individual cats with 2 VPT-treated teeth could experience both success and failure after VPT on different teeth at the same time, suggests that tooth-specific and procedural variables alongside host factors may influence outcomes, underscoring the need for case-by-case clinical assessment.

Imaging modalities differed among cases, with CBCT providing potentially greater sensitivity in detecting apical pathology than traditional dental radiographs.^28^ This variability may have influenced outcome classifications in cases where only dental radiography was performed. Notably, a human dentistry study showed that favorable outcomes were observed significantly less frequently when evaluated by CBCT than by dental radiography. In their study of indirect pulp capping with calcium silicate materials, 72 molar teeth diagnosed with reversible pulpitis were examined using both modalities at baseline and 12 months post-treatment. The proportion of cases deemed successful (ie, no signs of periapical radiolucency) at follow-up was 90% when judged by dental radiographs, but only 65% when assessed with CBCT.^29^ Another human study showed that periapical radiolucencies were present in 38.8% of roots when assessed by dental radiography, and in 57.6% of roots when assessed by CBCT.^28^ Incorporation of CBCT as a routine part of long-term follow-up protocols may yield more accurate assessment of treatment success and failure, although accessibility and cost considerations remain.

The clinical implications of restoration length and quality remain uncertain as failures were seen with both short (2 mm) and standard (3-4 mm) restorations (Table 3), and only a minority of failed teeth had demonstrable restoration voids at the time of VPT, challenging preconceptions regarding the criticality of ideal restoration morphology. A study on VPT in dogs similarly found no association between restoration voids and treatment outcome.^15^

Failure rates were highest among teeth with CCF and contaminated pulps. Prompt treatment of CCF is considered critical to improve the likelihood of success in preserving pulp vitality and preventing progression to pulp necrosis or periapical disease.^3^ There is conflicting evidence in the literature regarding the effect of time from pulp exposure to treatment. A study on VPT in dogs found no significant association between time to treatment and outcome in CCF cases, with 1 successful VPT case being performed 250 hours after exposure.^15^ Another study reported 36-month follow-up outcomes after VPT for 97 mature canine teeth in dogs with CCF. Radiographically, 88.2% of teeth remained vital when treated within 48 hours, compared to 41.4% when treated within 1 week, and 23.5% when treated within 3 weeks of pulp exposure.^17^ In yet another study, 4 teeth with CCF in dogs were treated with VPT more than 168 hours after pulp exposure, and all 4 cases failed.^16^ In the present study, 5 of the teeth with CCF that were deemed successful had VPT performed within 48 hours, and 9 teeth with CCF and successful outcomes had VPT done between 60 and 96 hours after the CCF occurred, indicating that VPT can still be effective up to and maybe even longer than 96 hours after CCF in cats. This suggests that while earlier intervention is preferred, some delayed treatments may still yield favorable outcomes, though the risk of failure may increase with time. It should also be considered that the trauma leading to tooth fracture may induce pulpitis, which can ultimately result in loss of pulpal vitality.^14^

Antibiotic usage was prevalent, but its necessity remains unclear, highlighting a need for further investigation given concerns about antimicrobial resistance.^30^ Interestingly, 1 of the cats that did not receive antibiotics had VPT performed on 2 teeth of which 1 was deemed successful and the other 1 a failure.

An aspect still insufficiently characterized in feline dentistry is postoperative pain following VPT. In addition, in the present study, 19% of feline VPT cases resulted in failure, which may have resulted in long-term discomfort or pain. In human dentistry, mild pain within the first 24 hours after pulpotomy or partial pulpotomy is commonly reported but typically subsides within 7 days.^31^ Comparable studies in veterinary patients are lacking, and the assessment of oral pain in cats remains difficult.^25,26,32^ It is therefore possible that cats experience postoperative discomfort similar to humans, underscoring the importance of controlling pain after VPT. Analgesic treatment should be considered during the initial postoperative period. Nevertheless, maintaining pulp vitality and minimizing surgical trauma may reduce the risk of both acute and chronic oral pain.

In young cats with immature permanent teeth, VPT may be particularly valuable due to the potential for continued root development and apical closure. In this study, 7 out of 10 teeth with open apices at the time of VPT demonstrated apical closure during follow-up, highlighting the capacity of VPT to enable apexogenesis in young feline patients. This finding aligns with current endodontic principles in human dentistry emphasizing the importance of preserving pulp vitality in immature permanent teeth to enable natural root development.^19^ Long-term radiographic monitoring is essential in these cases to confirm progressive apical closure and detect any complications early. Even though not significant in this study, the demonstrated apical closure in a majority of cases in this cohort supports the recommendation of VPT for young cats with recent CCF or malocclusion to optimize both tooth vitality and structural integrity.

In this study, TR was identified as one of the causes of failure in a small number of VPT-treated teeth, including a case where a tooth initially classified as successful developed TR type 2 and required intentional crown amputation after 4 years. It cannot be definitively determined whether TR observed in some treated teeth was caused by the VPT procedure itself or if these teeth would have developed TR regardless, given the high prevalence of this disease in cats.^33,34^ This uncertainty highlights the need for careful long-term radiographic follow-up in feline patients after VPT to distinguish treatment-related complications from independent dental pathology, and to manage TR appropriately if it arises.

All teeth in the study received MTA as a pulp dressing material, which has shown great biocompatibility, sealing ability and success in veterinary and human dentistry,^23,35–37^ possibly contributing to the positive outcome. Other pulp dressing materials may yield different results.

In the present study, secondary dentin formation was observed in the majority of treated teeth during follow-up, indicating survival of the pulp and tooth after VPT.^20,38^ However, in some cases, particularly older cats or those with shorter follow-up periods, secondary dentin formation was difficult to detect radiographically due to the naturally reduced pulp space, projection angle of the radiographs or limited time for the secondary dentin to build up. In addition, radiographic signs may lag behind microscopic changes.^21^

The role of tertiary dentin bridge formation as an indicator of treatment success after VPT in cats remains unclear. One study evaluated the pulp reaction in 32 feline teeth treated with VPT.^22^ Histologic examination 4 months post-treatment revealed dentin bridge formation in 27 teeth. Signs of pulp necrosis were reported in 14 teeth.^22^ A clinical study of 190 teeth treated with VPT in dogs found that formation of a tertiary dentin bridge was not significantly associated with increased or decreased odds of treatment failure.^15^ In human dentistry, reparative dentin can be deposited in a pulp that is irreversibly injured and its presence does not necessarily signify a favorable prognosis.^27^

VPT offers several advantages over conventional root canal treatment. The procedure is generally faster to perform and requires fewer instruments as well as smaller amounts of restorative material. In addition, VPT preserves the vitality of the tooth, allowing continued physiological function, dentin deposition, and maintenance of pulpal health. This preservation of vitality may contribute to improved long-term outcomes and reduced treatment complexity compared to non-vital approaches. These characteristics make VPT an appealing alternative to root canal therapy, particularly in cases where maintaining tooth vitality is feasible and beneficial for the patient's overall oral health.^39^ However, not all teeth are suitable candidates for VPT; in such situations, root canal treatment or extraction may be more appropriate treatment options.^13^

In patients with head trauma, studies recommend extraction^40^ or root canal therapy of fractured teeth with CCF.^41^ In select cases, VPT may be considered a treatment option to minimize the duration of anesthesia required compared to conventional root canal treatment. Moreover, extraction procedures require holding and manipulating the head, which may increase the risk of further injury in such cases. Importantly, all patients with recent head trauma should be stabilized before undergoing general anesthesia.

VPT, especially in feline teeth, due to their small size, is a technique-sensitive procedure that demands specific materials and practitioner expertise to achieve optimal outcomes.^13^ Hence, veterinary dentists performing VPT should have adequate training and experience, and ongoing education is recommended to maintain proficiency with evolving materials and techniques.

### Limitations and Future Studies

The lack of statistical significance between treatment outcomes and any of the analyzed variables is likely due to the relatively small sample size and limited number of failure cases, which reduces the study's power to detect meaningful differences. Although trends and clinical observations provide valuable insights, the results should be interpreted with caution.

As mentioned, a limitation of the present study was the relatively small sample size, partly explained by a low proportion of cases with adequate follow-up—only 47 of 112 treated teeth (42%) in 36 of 92 cats (39%). This can be compared to the follow-up rates reported in 2 previous studies on feline root canal therapy, where 80.5% of patients (37 of 46 cats)^42^ and 57% (32 of 56 cats)^43^ returned for evaluation. The limited follow-up rate in the current study is likely multifactorial. Contributing factors may include referral-based patient flow, owners’ perception of their cat's well-being (potentially reducing motivation for re-examination), delegation of follow-up responsibilities to other practitioners, and mortality linked to unrelated diseases. Future research could benefit from implementing structured recall systems or standardized follow-up protocols, potentially supported by systematic reminders to both owners and referring veterinarians. In addition, early detection of potential problems is crucial for the well-being of the cat.

Other limitations include potential observer bias due to single-operator image evaluation and lack of histopathological confirmation. Nevertheless, to the authors’ knowledge, this is the largest feline VPT study reported to date, providing useful insight on clinical outcomes.

Further research is needed to optimize treatment and follow-up protocols, define prognostic indicators, and clarify the role of adjunctive therapies such as antibiotics and advanced imaging.

## Conclusion

In conclusion, this study shows a relatively high success rate of VPT in cats and suggests VPT as a viable alternative to extraction or root canal therapy in select feline cases such as recent CCF and traumatic malocclusion, offering advantages in preserving tooth structure and function. The presence of late failures underscores the importance of long-term, ideally lifelong, annual clinical and radiographic monitoring to identify and manage potential complications in a timely manner.

## Materials

- DEHP Bur Diamond FG 001-009M, Henry Schein, Hamburg, Germany.
- Saline 0.9% NaCl, Braun Aesculap Chifa Sp. z o.o. Nowy Tomysl, Poland.
- ProRoot MTA^®^, Dentsply Sirona, Konstanz, Germany.
- Ionoseal, VOCO GmbH Cuxhaven, Germany.
- Herculite XRV, Nobel Biocare Services AG – Kerr.
- Business Balz Zimmermann-Strasse 7, 8302 Kloten, Switzerland. Sanding disc, Sof-Lex, 3M ESPE, St. Paul, MN, USA.
- Scotchbond Universal Adhesive, 3M ESPE, Seefeld, Germany.
- Lidocainym hydrochloricum WZF 2%, Polfa S.A. Warszawskie zakłady farmaceutyczne, Warszawa, Poland.
- Metacam^®^, Boehringer Ingelheim Vetmedica Sp. z o.o., Warsaw, Poland.
- Bupredine Multidose 0.3 mg/ml, Dechra Veterinary Products sp. z o.o. Warszawa, Poland.
- Image Plate Scanner, CR7 Image Plate Plus Size 2, Dürr Medical, Germany.
- Vet-Exam Pro Ink Version 1.2.0, Dürr NDT GmbH & amp, Co. KG, Bietigheim Bissingen, Germany.
- Progeny^®^ dental xray unit, Midmark Corporation, USA.
- NewTom 5G XL^®^, QR s.r.l., Verona, Italy.
- NNT^®^ Version 10.1, QR s.r.l., Verona, Italy.
- Convenia^®^, Zoetis Poland Sp. z o.o., Warsaw, Poland.
